# Psychological distress in Spanish-speaking countries during the COVID-19 pandemic: A systematic review and meta-analysis

**DOI:** 10.1097/MD.0000000000047062

**Published:** 2026-01-09

**Authors:** Kenny Escobar-Segovia, Sara Domínguez-Salas, Juan Jesús García-Iglesias, Daniel López-López, Regina Allande-Cussó, Carlos Ruiz-Frutos, Alejandro Artero-García, Juan Gómez-Salgado

**Affiliations:** a Faculty of Engineering in Earth Sciences – FICT, ESPOL Polytechnic University (ESPOL), Guayaquil, Ecuador; b Departamento de Psicología Experimental, Universidad de Sevilla, Sevilla, Spain; c Department of Sociology, Social Work and Public Health, Faculty of Labour Sciences, University of Huelva, Huelva, Spain; d Research, Health and Podiatry Group, Department of Health Sciences, Faculty of Nursing and Podiatry, Industrial Campus of Ferrol, University of A Coruña, Ferrol, Spain; e Department of Nursing, University of Seville, Seville, Spain; f Safety and Health Postgraduate Programme, Universidad Espíritu Santo, Guayaquil, Ecuador; g Department of Emergency, Ceuta University Hospital, Ceuta, Spain.

**Keywords:** COVID-19, mental health, psychological distress, Spanish-speaking countries

## Abstract

**Background:**

Psychological distress (PD) has increased significantly during the coronavirus disease 2019 (COVID-19) pandemic. In Spanish-speaking countries, with their cultural, social, and economic diversity, this phenomenon has become particularly relevant and has been aggravated by factors such as socioeconomic inequalities and unequal access to mental health services. The aim of this systematic review was to consolidate the available knowledge on PD in Spanish-speaking population groups by assessing both the prevalence of symptoms and the associated factors in different demographic groups and geographic contexts, during the COVID-19 pandemic.

**Methods:**

A systematic review following the PRISMA (Preferred Reporting Items for Systematic reviews and Meta-Analyses) statement was conducted in the Web of Science, PubMed, and Scopus electronic databases in January 2025. The search included studies published from the beginning of the pandemic until May 2023. The Joanna Briggs Institute’s critical assessment tool was used to evaluate the chosen studies’ methodological quality.

**Results:**

A total of 53 studies were included in the review, which involved research conducted in Spain, Peru, Chile, Ecuador, Argentina, and Colombia. The results revealed a high prevalence of PD in these countries, especially among healthcare workers, women, and young people. The assessment methods used included the General Health Questionnaire (GHQ, GHQ-12, and GHQ-28 versions), the Kessler scale (K-6 and K-10 versions), and the 90-symptom checklist questionnaire (SCL-90-R), that allowed obtaining various dimensions of PD. The studies also highlighted the importance of the sense of coherence and work engagement as protective factors.

**Conclusions:**

In the COVID-19 pandemic, PD was analyzed to be severe in Spanish-speaking countries, pointing to the need for specific and culturally adapted interventions to address this public mental health crisis. This is why public health policies must focus on the prevention and treatment of PD, with special attention to the most vulnerable groups.

## 1. Introduction

Psychological distress (PD) is a complex construct that encompasses a variety of emotional and cognitive symptoms, such as anxiety, depression, and stress, which negatively affect people’s overall well-being.^[1,2]^ This phenomenon has caught the attention of researchers and healthcare professionals worldwide given its increasing prevalence and substantial impact on the mental and physical health of the different population groups.^[3–5]^ Identifying factors that contribute to PD is essential for the development of effective interventions and public health policies, as is an understanding of its manifestations and consequences.^[1,6]^

In this sense, the labor demands and resources (JD-R) model developed by Demerouti et al^[7]^ proposes that the worker, in his relationship with work, has a series of work resources that, if valued positively, can help achieve the objectives of the job, reduce work demands, or stimulate personal growth and development.^[8]^ Likewise, the worker in his professional practice is exposed to a series of work demands that, if assessed negatively, can subject the worker to sustained physical or psychological stress.^[9]^ Variables such as sense of coherence (SOC) or work engagement (WE) can be elements of personal resources that the worker has to cope with the demands or stress of their daily practice.

In Spanish-speaking countries, where there is significant cultural, social, and economic diversity, PD has become a major issue. These nations, which range from Latin America^[10–12]^ to Spain,^[13,14]^ exhibit diverse sociocultural contexts that influence the way in which PD is experienced and managed. Factors such as economic inequalities, violence, migration, and differences in access to mental health services may play a key role in the prevalence and management of PD in these regions.^[15]^

In the countries studied, cultural, social, and economic differences significantly influence the way in which PD is presented and treated. For example, high levels of economic inequality and a lack of mental health services have led to greater prevalence of PD, especially among vulnerable populations like women and young people, in various Latin American nations like Peru and Ecuador.^[16,17]^ On the contrary, the high population density of countries like Spain and the strict confinement measures during the epidemic have worsened symptoms of anxiety and depression, even with better health infrastructure.^[13]^ These differences highlight the need to tailor interventions to address the socioeconomic and cultural specificities of each context. For example, community-based programs like “Acceso Salud Mental” in Chile have proven effective by providing psychosocial support in marginalized communities, illustrating how culturally adapted strategies can significantly impact PD management.^[4,18]^

However, research on PD in Spanish-speaking countries has been fragmented, focusing narrowly on very specific populations or local contexts. This sectoral approach makes it difficult to achieve a comprehensive understanding of PD across the region. This result in difficult development of intervention strategies that are culturally relevant and effective on a broader regional scale.^[19]^

The coronavirus disease 2019 (COVID-19) pandemic has aggravated contributing conditions to PD, and several recent studies have pointed to a significant increase in symptoms of anxiety, depression, and stress in different Spanish-speaking populations.^[20–22]^ This increase highlights the urgent need to better understand risk factors and people’s responses to extreme stress in these specific contexts. In addition, the pandemic has underlined inequalities in access to mental health care, with vulnerable groups being particularly affected.^[23,24]^

In this context, a systematic review is needed to compile and analyze the existing literature on PD in Spanish-speaking countries. This approach will identify common and different patterns in prevalence, risk factors, and interventions reported in the existing literature. Likewise, it will provide a solid evidence base to guide future research and formulate culturally effective and efficient mental health policies.

A contextualized and culturally sensitive approach to mental health research and practice is crucial, and this study makes a substantial contribution to our understanding of PD in Spanish-speaking nations. Additionally, it is anticipated that the results of this systematic analysis will help shape more inclusive mental health policies that address the unique need of Spanish-speaking populations in the wake of the epidemic.

Across the globe, experiences with personal development during the COVID-19 pandemic have demonstrated considerable differences among regions. In Asia, for instance, nations like China adopted early mental health strategies utilizing digital resources, including mobile apps and telepsychology services, which alleviated some effects of lockdowns on the wider community.^[5]^ In North America, socioeconomic discrepancies in the treatment of PD were brought to light by unequal access to mental health treatments in the United States.^[6]^ Comparing these approaches with those of Spanish-speaking countries reveals not only the need to strengthen local capacities but also the opportunity to learn from strategies implemented in other cultural contexts. This comparative analysis underscores the relevance of this study by positioning it as a key contribution to identifying regional patterns and transferable strategies for managing PD.

Therefore, the main objective of this systematic review was to consolidate the available knowledge on PD in Spanish-speaking population groups by assessing both the prevalence of symptoms and the associated factors in different demographic groups and geographic contexts, during the COVID-19 pandemic.

## 2. Methods

### 2.1. Study design

A systematic review was conducted following the guidelines of the Preferred Reporting Items for Systematic reviews and Meta-Analyses (PRISMA) statement.^[25]^ The authors relied on a protocol to carry out this systematic review, which was registered in the International Prospective Register for Systematic Reviews (PROSPERO) of the University of York, with identification code CRD42024580489. Given that our study focused on systematic review and meta-analysis, there was no necessity for ethical review board approval or obtaining informed consent from the participants.

### 2.2. Databases and search strategy

To manage and synthesize the information, software tools such as Mendeley were used for reference management, and Microsoft Excel was employed for data organization and preliminary analysis. The search was carried out in the PubMed, Scopus, and Web of Science electronic databases on the basis of the key words that the research question yielded, following the Condition, Context, Population strategy (Table 1).

**Table 1:** CoCoPop format.

Condition (Co)	Psychological distress, measured through validated self-report instruments
Context (Co)	COVID-19 pandemic
Population (Pop)	Adults (≥18 years old) residing in Spanish-speaking countries, including general population, students, employees, and healthcare professionals.
Research question	*What is the prevalence of psychological distress during the COVID-19 pandemic in adult populations residing in Spanish-speaking countries, as assessed by validated self-report instruments*?

CoCoPop = Condition, Context, Population.

Based on these key words, the Medical Subject Headings thesaurus was consulted and the corresponding descriptors were obtained. In order to improve the capture of published studies in line with the subject of the review, synonymous terms capable of completing the search strategy were used to complete the search based on the Medical Subject Headings descriptors (Table 2), joined by means of the Boolean operators *AND OR*.

**Table 2:** Terms used in the search.

MeSH	Terms
Latin America	Hispanic*, Latino, Argentina, Argentine, Bolivia, Bolivian, Chile, Chilean, Colombia, Colombian, Costa Rica, Costa Rican, Cuba, Cuban, Dominican Republic, Dominican, Ecuador, Ecuadorian, El Salvador, Salvadoran, Guatemala, Guatemalan, Honduras, Honduran, Mexico, Mexican, Nicaragua, Nicaraguan, Panama, Panamanian, Paraguay, Paraguayan, Peru, Peruvian, Puerto Rico, Puerto Rican, Uruguay, Uruguayan, Venezuela, Venezuelan
Spain	Spain, Spanish
Psychological distress	Psychological distress, emotional distress
Surveys	Survey*, questionnaire*, scale*, instrument*

* Truncation wildcard to retrieve all variants of a word from its common root.

Table 3 shows the search strategy used, carried out on January 5, 2025, for each of the above databases during the search process.

**Table 3:** Search strategy and databases.

Database	Search strategy	Date of search	Results
PubMed	(((((Hispanic or Latino[MeSH Terms]) OR (Latin America[MeSH Terms])) OR (Hispanic*[Title/Abstract] OR Latin America*[Title/Abstract] OR Argentina[Title/Abstract] OR Argentine[Title/Abstract] OR Bolivia[Title/Abstract] OR Bolivian[Title/Abstract] OR Chile[Title/Abstract] OR Chilean[Title/Abstract] OR Colombia[Title/Abstract] OR Colombian[Title/Abstract] OR Costa Rica[Title/Abstract] OR Costa Rican[Title/Abstract] OR Cuba[Title/Abstract] OR Cuban[Title/Abstract] OR Dominican Republic[Title/Abstract] OR Dominican[Title/Abstract] OR Ecuador[Title/Abstract] OR Ecuadorian[Title/Abstract] OR El Salvador[Title/Abstract] OR Salvadoran[Title/Abstract] OR Guatemala[Title/Abstract] OR Guatemalan[Title/Abstract] OR Honduras[Title/Abstract] OR Honduran[Title/Abstract] OR Mexico[Title/Abstract] OR Mexican[Title/Abstract] OR Nicaragua[Title/Abstract] OR Nicaraguan[Title/Abstract] OR Panama[Title/Abstract] OR Panamanian[Title/Abstract] OR Paraguay[Title/Abstract] OR Paraguayan[Title/Abstract] OR Peru[Title/Abstract] OR Peruvian[Title/Abstract] OR Puerto Rico[Title/Abstract] OR Puerto Rican[Title/Abstract] OR Uruguay[Title/Abstract] OR Uruguayan[Title/Abstract] OR Venezuela[Title/Abstract] OR Venezuelan[Title/Abstract] OR Spain[Title/Abstract] OR Spanish[Title/Abstract])) AND (((Psychological Distress[MeSH Terms]) OR (“Psychological Distress”[Title/Abstract])) OR (“Emotional Distress”[Title/Abstract]))) AND (((((Surveys and Questionnaires[MeSH Terms]) OR (Survey*[Title/Abstract])) OR (Questionnaire*[Title/Abstract])) OR (scale*[Title/Abstract])) OR (instrument*[Title/Abstract]))) AND ((COVID-19[MeSH Terms]) OR (COVID-19[Title/Abstract] OR SARS-CoV-2[Title/Abstract]))	January 5, 2025	224
Scopus	(TITLE-ABS-KEY (hispanic* OR “Latin America*” OR argentina OR argentine OR bolivia OR bolivian OR chile OR chilean OR colombia OR colombian OR “Costa Rica” OR “Costa Rican” OR cuba OR cuban OR “Dominican Republic” OR dominican OR ecuador OR ecuadorian OR “El Salvador” OR salvadoran OR guatemala OR guatemalan OR honduras OR honduran OR mexico OR mexican OR nicaragua OR nicaraguan OR panama OR panamanian OR paraguay OR paraguayan OR peru OR peruvian OR “Puerto Rico” OR “Puerto Rican” OR uruguay OR uruguayan OR venezuela OR venezuelan OR spain OR spanish) AND TITLE-ABS-KEY (“psychological distress” OR “emotional distress”) AND TITLE-ABS-KEY (survey* OR questionnaire* OR scale* OR instrument*) AND TITLE-ABS-KEY (COVID-19 OR SARS-CoV-2))	January 5, 2025	304
Web of Science	(((ALL = (Hispanic* OR “Latin America*” OR Argentina OR Argentine OR Bolivia OR Bolivian OR Chile OR Chilean OR Colombia OR Colombian OR “Costa Rica” OR “Costa Rican” OR Cuba OR Cuban OR “Dominican Republic” OR Dominican OR Ecuador OR Ecuadorian OR “El Salvador” OR Salvadoran OR Guatemala OR Guatemalan OR Honduras OR Honduran OR Mexico OR Mexican OR Nicaragua OR Nicaraguan OR Panama OR Panamanian OR Paraguay OR Paraguayan OR Peru OR Peruvian OR “Puerto Rico” OR “Puerto Rican” OR Uruguay OR Uruguayan OR Venezuela OR Venezuelan OR Spain OR Spanish)) AND ALL=(“psychological distress” OR “emotional distress”)) AND ALL = (Survey* OR Questionnaire* OR scale* OR instrument*)) AND ALL = (COVID-19 OR SARS-CoV-2)	January 5, 2025	390
Total			918

* Truncation wildcard to retrieve all variants of a word from its common root.

### 2.3. Selection criteria

The following criteria were used for the selection of the articles:

Inclusion criteria:

- Original articles published in English, Spanish, French, and Portuguese.
- Type: original articles, meta-analysis, short communications, and case reports.
- Full-text articles.
- Peer-reviewed articles.
- Studies where data collection period was from March 11, 2020 to May 5, 2023.
- Articles measuring any of the following values and/or effects: PD or distress in people from Spanish-speaking countries, with specific validated questionnaires.

Exclusion criteria:

- Studies of low scientific-technical quality after applying the quality assessment tool, if they did not meet at least 6 out of the 9 criteria on the Joanna Briggs Institute (JBI) checklist.
- Articles that did not respond to the research question and were not related to the objective of the review.
- Type: systematic reviews, opinion pieces, editorials, and letters to the editor.

### 2.4. Data collection and extraction

Two researchers independently conducted searches, eliminated duplicate studies, and selected articles for inclusion after reading the abstract and title according to previously established criteria. Subsequently, the same 2 authors reviewed the full text of the studies potentially eligible for inclusion in the review and the decision to include or exclude them was made by consensus, and discrepancies were resolved by a third author.

### 2.5. Methodological quality assessment

To mitigate publication bias, exhaustive searches were conducted in multiple international databases. Two reviewers independently ascertained the methodological quality of the selected studies using the critical appraisal tools of the JBI at the University of Adelaide.^[26,27]^ These tools can be used to assess the methodological quality of a study and to determine the extent to which a study has excluded or minimized the possibility of bias in its design, conduct, and/or analysis. The versions for quantitative cross-sectional studies (8 items) and for qualitative and longitudinal studies (11 items) were used, with a cutoff point of 6 for inclusion in this review (see Tables S1 and S2, Supplemental Digital Content, https://links.lww.com/MD/R105).

### 2.6. Data analysis

A random-effects meta-analysis was performed using the meta-analysis package of StatsDirect software (4.0.4 version; Wirral, England, United Kingdom) to estimate the prevalence of PD in Spanish-speaking population groups during the COVID-19 pandemic (workers, healthcare workers, and general population). To estimate the prevalence, the total sample size and the sample with PD of each study were used. A random-effects model meta-analysis was chosen to calculate mean prevalence rates and 95% confidence interval (CI) because the number of included studies was higher than 10. Heterogeneity was calculated using the *I*^2^ index and publication bias using Egger test.

## 3. Results

**Figure 1.**
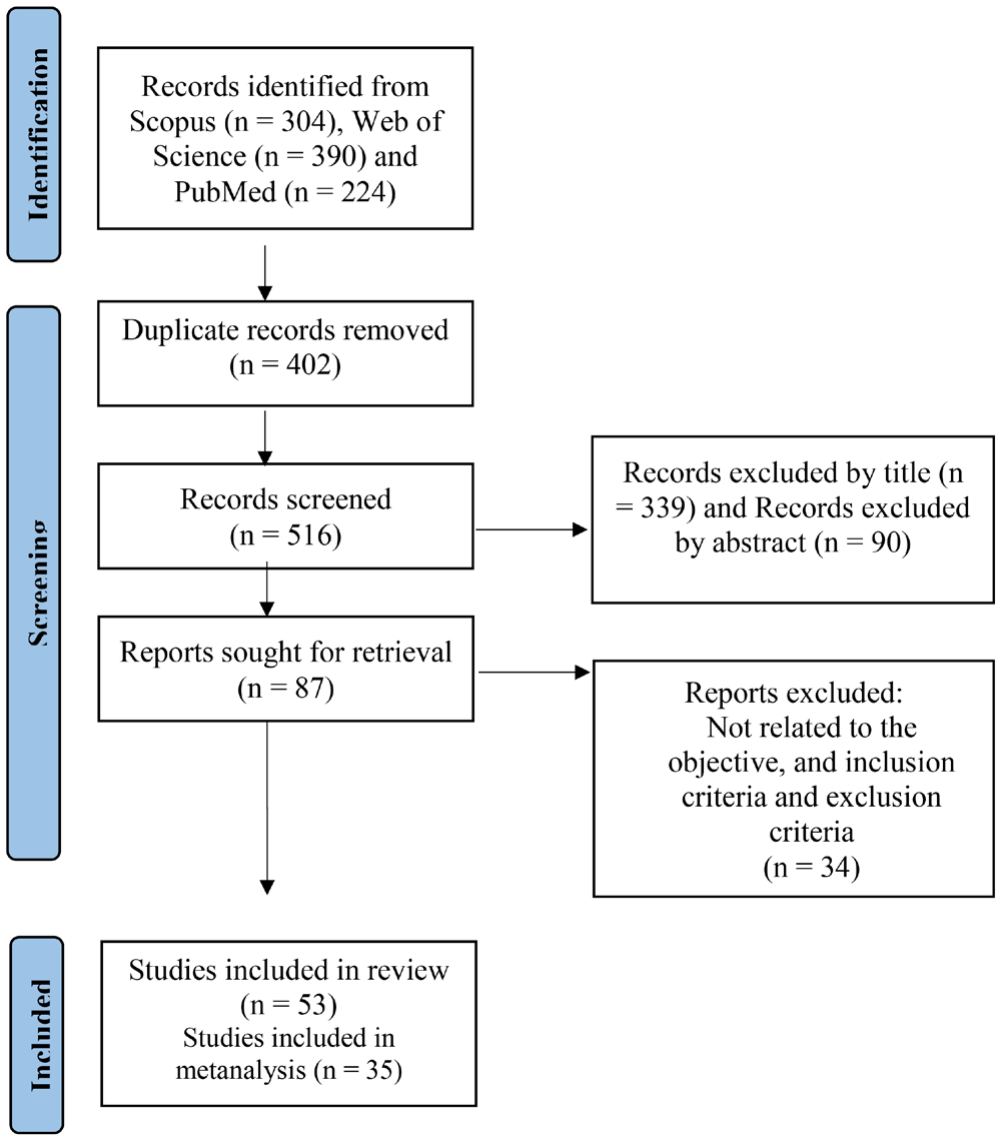
Search results (flowchart—PRISMA). PRISMA = Preferred Reporting Items for Systematic reviews and Meta-Analyses.^[25]^

The initial search strategy identified a total of 874 references, which were then screened according to the topic of this review. A total of 53 studies were finally selected (Fig. 1), 50 of them cross-sectional, and 3 longitudinal or cohort studies. In the PRISMA flowchart, out of the 53 studies in the systematic review, 35 remained in the meta-analysis for 2 reasons: some studies used the same sample (n = 9), and in 9 cases, prevalence was not detailed, and the authors did not respond to the information request email.

Of the 53 studies included in the review,^[4,11,13,14,16–18,28–74]^ 35 had been conducted in Spain, 6 in Peru, 3 in Ecuador, 3 in Argentina, 3 in Chile, 2 in Colombia, and 1 study included samples from 2 countries, Colombia and Spain. The included studies were assessed with the JBI critical appraisal tool, with both cross-sectional observational studies and longitudinal or cohort studies scoring medium-high. Table 4 is based on the Iberoamerican Cochrane Centre Handbook and it shows the characteristics of each of the studies included in this review. These were classified by authors and year of publication, country, design and objective, participants, instrument, and main results; in addition, the results of the JBI critical appraisal tool were added.

**Table 4:** Characteristics of the studies included in the systematic review.

Author and country	Date of data collection	Study objective	Type of study	Participants	Methods	Main findings	Quality of the studies
Pilar Matud et al^[37]^ Spain	April–June 2021	To assess PD on a sample of unemployed migrants residing in informal settlements in the province of Huelva (southern Spain), during the fourth wave of the COVID-19 pandemic.	Cross-sectional	317 unemployed migrants	GHQ-12	The mean PD score found in unemployed immigrants living in informal settlements in Huelva indicated high levels of PD (GHQ-12 = 13). Among the participants, those who had experienced isolation due to contact with COVID-19, used substances, and those with chronic illnesses reported the highest levels of PD.	6/8
Carranza Esteban et al^[38]^ Spain	October–December 2020	To investigate gender differences in COVID-19-related stressful events, PD, and well-being during the second wave of the COVID-19 pandemic in Spain.	Cross-sectional	1758 participants	SPANE SWLS BIT GHQ-12 RSES SSS	More than half of the sample (56.5%) had scores that exceeded the cutoff point to be classified as PD, and this percentage was higher in women (63.4%) than in men (49.5%).	8/8
Ripoll et al^[13]^ Spain	April–June 2020	To explore (1) the mental health impact on HCWs in the Balearic Islands during the initial months of the pandemic, (2) the influencing factors, (3) and the experiences of those in a COVID-19 environment.	Cross-sectional	336 HCWs	GHQ-28 PTSD	55.2% had PD (GHQ-28 scale) and 27.9% had worse PTSD symptoms (DTS scale).	6/8
Higgins and Green^[35]^ Colombia and Spain	June–November 2021	To identify factors related to mental health disorders in undergraduate nursing students during the COVID-19 pandemic.	Cross-sectional	302 students from Spain and Colombia	GHQ-12 APGAR MOS IES-R	187 students were found to manifest clinically significant mental health problems, with a prevalence of 61.92 % according to the General Health Questionnaire (GHQ-12) and a mean score of 13.8 (SD = 6.7). No statistical differences were found between the prevalence of these problems across countries (58.67% for Spain and 63.00% for Colombia, *P* = .503) nor in mean GHQ-12 scores (Spain 13.52 ± 5.4 and Colombia 13.93 ± 7.04).	7/8
Gómez-Salgado et al^[39]^ Peru	August–September 2020	To determine whether concern about COVID-19 and workloads predict PD in HCWs.	Cross-sectional	367 HCWs	K-6 EPPC-Cov19 ECT	Concern about COVID-19 (β = –0.436; *P* < .01) and workload (β = 0.239; *P* < .01) are variables that significantly predict psychological discomfort (adjusted *R*^2^ = 0.33).	6/8
Ochagavía-Berasategui et al^[40]^ Spain	March–May 2022	To evaluate the level of PD among construction workers in an advanced phase of the COVID-19 pandemic in Andalusia, southern Spain.	Cross-sectional	860 participants	EIQ COVID-19 GHQ-12	The prevalence of PD (GHQ < 3) among workers was 29.2%, with a higher incidence in women (37.7%) compared to men (27.3%) (*P* = .010). Participants with PD were younger (mean = 41.2; SD = 10.4) than those without PD (mean = 43.4; SD = 10.3) (*P* = .006). Subjects with PD had lower levels of health and a worse physical condition (*P* < .001).	6/8
Andújar-Barroso et al^[41]^ Spain	February–December 2022	To analyze the number and type of emergencies, distress, and fear of getting infected among brackets and aligners orthodontic patients during the COVID-19 lockdown.	Cross-sectional	324 participants	K-10	The study revealed that 21% of patients had PD associated with orthodontic treatment. There were no differences among brackets or aligner patients.	6/8
Gómez-Salgado et al^[32]^ Chile	April–December 2020	To analyze the relationship between SOC, WE, and work environment variables as predictors of the level of PD during the first phase of the COVID-19 pandemic in Chile.	Cross-sectional	1466 participants	UWES-9 SOC-13 GHQ-12	72.7% of participants had high levels of PD, more predominantly among women, with work stress and low SOC acting as the most influential mediators in generating PD, and even more so when both were combined. Low WE and the availability of safe and effective means to prevent infection were predictors of PD among workers.	6/8
Dorado Barbé et al^[42]^ Spain	April–June 2021	To assess the presence of anxiety, fear, and PD in the population of people over 65 years of age and to study possible differences with a sample of subjects aged between 60 and 65 years.	Cross-sectional	1112 participants	AMICO GHQ-12	Levels of anxiety and fear of COVID-19 in older people in Spain are moderate, and PD is higher than in the general population. Women over 60 years of age, and those over 65 years of age, have higher values of both general distress and fear and anxiety of COVID-19 than their male counterparts. In the specific case of people over 65 years of age, women who had not experienced COVID-19 and were single or widowed had higher levels of PD, fear, and anxiety. Furthermore, women had 0.10 times more anxiety and fear of COVID-19 and 0.19 times more emotional distress than men.	8/8
Matud et al^[43]^ Spain	April 2020	To analyze the potential relationship that may exist between social and personal factors and the levels of PD experienced by the Spanish population during lockdown.	Cross-sectional	3436 participants	K-10	Gender has a statistically significant impact on PD. Females show higher levels of PD.	8/8
Gómez-Salgado et al^[44]^ Spain	February–April 2022 and October 2022–February 2023	(i) To determine the existence of differences between women and men in PD; cognitive and affective components of subjective well-being; and perceived vulnerability to diseases, resilience, self-esteem, and social support 2 years after the onset of the COVID-19 pandemic and at the end of the first wave of the pandemic, and (ii) to assess the relevance of sociodemographic characteristics and perceived vulnerability to diseases, resilience, self-esteem, and social support on PD and subjective well-being in women and men 2 years after the onset of the COVID-19 pandemic and at the end of the first wave of the pandemic.	Cross-sectional	1588 participants	GHQ-12 SPANE SWLS PVDQ BRS RSES SSS	At Time 1 (February–April 2022), more than half of the women (57.4%) had scores that exceeded the threshold for distressed cases, whereas the percentage for men totaled 38.7%, showing statistically significant differences, χ^2^ (1, N = 460) = 16.10, *P* < .001. Time 2 (from October 2022 to February 2023), classified half of the women, 50.5%, and 41.5% of the men as psychologically distressed, also configuring statistically significant differences.	6/8
Ames-Guerrero et al^[45]^ Ecuador	April–May 2020	To test the association between the SOC, WE, and PD in HCWs in Ecuador during the first phase of the COVID-19 pandemic.	Cross-sectional	803 HCWs	UWES-9 GHQ-12 SOC-13	There is a positive correlation (*P* < .01) between the SOC and WE, and a negative correlation with PD. The mean value for PD was *M* = 4.58.	8/8
Alaminos-Torres et al^[46]^ Peru	March–April 2020	To broaden current knowledge about active or passive coping strategies and how they are linked to psychological health in the general population exposed to the COVID-19 pandemic in Peru.	Cross-sectional	434 participants	GHQ-28 COPE-28	The positive correlation suggests an increase in somatic symptoms (*R* = 0.20), anxiety (*R* = 0.13), and social dysfunction (*R* = 0.15) among those with better strategy of emotional support. Moreover, greater anxiety/insomnia (*R* = 0.16) and social dysfunction (*R* = 0.13) among those with higher social support. The planning strategy correlates inversely with severe depression (*r* = − 0.19). Active strategies of positive reframing and humor do not correlate with any indicator measured by the GHQ.	6/8
Ruiz-Frutos et al^[16]^ Ecuador	2 April and May 17, 2020	To find the relationship between work environment factors and WE among the Ecuadorian general population during the first phase of the COVID-19 pandemic to assess their levels of PD.	Cross-sectional	2161 participants	UWES-9 GHQ-12	The variables that predicted 70.2% of PD during the first phase of the pandemic were being female, with a low level of vigor (WE dimension), being stressed at work, and low job satisfaction.	8/8
Bedoya Cardona et al^[33]^ Colombia	April 2020 and February 2021	To analyze the effects on mental health in 2 phases of the COVID-19 pandemic (April 2020 and February 2021) in the population of Colombia.	Longitudinal	1309 participants	GHQ-12	A higher level of PD was observed among women (*M* = 3.99, SD = 3.39) (*P* < .001), in those who lived without a partner (*M* = 3.83, SD = 3.47) (*P* = .036), and in those with a worse perception of health (*M* = 6.27, SD = 3.51) (*P* < .001). PD decreased in the second period from *M* = 3.99 (SD = 3.36) to *M* = 2.98 (SD = 3.30) (*P* < .001).	8/11
Mediavilla et al^[47]^ Spain	October 2020	To evaluate the PD of Spanish airline pilots, a group of professionals undergoing an unprecedented work situation as a result of the COVID-19 pandemic.	Cross-sectional	342 participants	GHQ-12	The total score on the standard GHQ-12 was 4.54 ± 3.31 very close to the cutoff point established to determine PD. The total proportion of individuals presenting PD risk was 43.3%.	8/8
Merino-Godoy et al^[48]^ Spain	April–June 2020	To explore the association between 3 work-related stressors (informed access to personal information, protective equipment, changes compared to usual work, decision making regarding patient prioritization practices) and (1) fear of contagion and (2) various mental health outcomes, including PD, depressive symptoms, and death thoughts.	Cross-sectional	1786 participants	C-SSRS PHQ-9 GHQ-12	It was estimated that 74% and 27% of respondents tested positive for PD and probable depression, respectively, and 7% reported suicidal desires. Respondents were more afraid of infecting their loved ones than of becoming infected themselves. All work-related stressors were associated with symptoms of depression and PD in adjusted models.	8/8
Ortiz-Calvo et al^[49]^ Spain	Second half of the year 2020/2021	To measure and analyze the association of emotional burnout and resilience with the PD of students who finished their nursing studies after the peak of the COVID-19 pandemic.	Cross-sectional	393 university students	K-10 EBS CD-RISC10	Last-year students of the degree of nursing obtained, on average, a higher score in resilience (*M* = 36.34) than in PD (*M* = 27.89) and emotional burnout (*M* = 26.28).	8/8
Domínguez-Salas et al^[50]^ Spain	April–June 2020	To study the potential effect of self-perceived social support and a resilience factor, namely the self-reported ability to bounce back, on the mental health outcomes of a large sample of HCWs from Spain during the initial COVID-19 pandemic outbreak.	Cross-sectional	2372 participants	BRS PHQ-9 GHQ-12	PD and depressive symptoms were more frequent among younger age groups, while death thoughts did not show a clear age pattern. Women reported depressive symptoms, PD, and death thoughts, more frequently than men. Education attainment was directly associated with reported PD. While physicians and nurses reported the highest scores overall in depressive symptoms, PD was reportedly more common among health technicians. Frontline HCWs reported distinctly higher presence of depressive symptoms, PD, and death thoughts.	8/8
Ruiz-Frutos et al^[51]^ Spain	March–April 2020	To analyze the relationship between WE, SOC, and PD levels in Spanish HCWs who were active during the COVID-19 pandemic lockdown.	Cross-sectional	1459 HCWs	UWES-9 SOC-13 GHQ-12	SOC and WE are protective factors against PD.	6/8
Martínez-Caballero et al^[52]^ Spain	April–June 2020	To assess PD of occupational HCWs and its relationship with their WE and work environment characteristics.	Cross-sectional	499 nurses and physicians	Self-elaborated Work Environment Questionnaire GHQ-12 UWES-9	A total of 65.53% of the participants had PD and the total mean score of the UWES-9 scale was 34.80 (SD = 10.69). Workload, conflicts, stressful situations, and less job satisfaction were significantly related to a higher percentage of PD (*P* < .05).	8/8
Tiga-Loza et al^[34]^ Colombia	April–June 2020	To analyze the perceived stress, coping strategies, emotional regulation, impact of the event, and PD during quarantine due to COVID-19 pandemic in the Colombian population.	Cross-sectional	356 participants	PSS COPE ERQ IES-R SCL-90-R	A comparative analysis of stress, the impact of quarantine, and PD according to sociodemographic characteristics found that women, people under 35 years of age, high school graduates, students, and living alone during the quarantine showed higher affectation. These differences were statistically significant, except for the impact of quarantine by sex and cohabiting partners.	6/8
Lucuix et al^[29]^ Ecuador	April–May 2020	To analyze the level of PD in the population of Ecuador during the first phase of the COVID-19 pandemic.	Cross-sectional	3640 participants	GHQ-12	As can be seen, in Table 2, 62.72% of the sample has PD, with a cutoff point of GHQ ≥ 3. The overall score on the 12 items is *M* = 4.41 (SD = 3.49). Women report a higher percentage of PD (69.1%) than men (55.0%), *P* < .001, OR = 0.546, 95% CI = 0.477–0.626.	6/8
Germano and Brenlla^[30]^ Argentina	May–August 2020	To analyze the PD in an Argentine population sample during the COVID-19 pandemic, identifying the potential predictive role of sociodemographic variables and factors related to the general health of the participants.	Cross-sectional	1112 participants	GHQ-12	60.9% of the sample presented PD. The variables that showed a predictive character about the presence of PD were sex, specifically being a woman, being of younger age, presenting a higher number of symptoms related to COVID-19, and a lower score in self-perceived health.	6/8
Mamani-Benito et al^[11]^ Peru	First 3 months of 2022	To determine the socio-occupational factors associated with PD in HCWs during the COVID-19 pandemic in the Peruvian highlands.	Cross-sectional	433 HCWs	K-6	When the multivariate analysis of socio-occupational factors was performed, PD was found to be associated with fear (those with a higher score of fear, 58.6%, and those with no fear, 25.7% of PD; *P* < .001) and with work overload (those with a higher perception of work overload, 47.6%, and those with no work overload, 26.5% of PD; *P* < .001).	6/8
Allande-Cussó et al^[53]^ Spain	May–July 2020	To analyze the impact of the COVID-19 pandemic on EMS professionals in terms of their mental health.	Cross-sectional	317 EMS workers	GHQ-12 DTS-8 AIS-8	36% of respondents had PD, 30.9% potentially had PTSD, and 60.9% experienced insomnia. Years of work experience were found to be positively correlated, albeit with low effect, with the PTSD score (*R* = 0.133).	8/8
Gómez-Salgado et al^[54]^ Spain	April–June 2021	To describe and evaluate the sociodemographic profile and assess the levels of anxiety and fear, WE, and PD on a sample of migrants living in settlements in the province of Huelva (Spain) during the COVID-19 pandemic.	Cross-sectional	623 migrants	AMICO UWES-9 GHQ-12	The mean score of the GHQ-12 questionnaire was 14.12 points (SD = 4.8), and this would indicate the possibility that the sample is suffering from PD. No significant differences were found between the presence of PD, as assessed by the GHQ-12 scale, and different values for education, work activity, work permit, route of entry to Spain, and desire to return to their country of origin.	8/8
Ruiz-Frutos et al^[4]^ Chile	April–December 2020	To present the effects of the first wave of the COVID-19 pandemic on the mental health of Chileans, in particular on the development of PD.	Cross-sectional	3227 participants	GHQ-12	78.83% of the sample had PD, following the ≥3 cutoff point of the GHQ-12. The overall mean of the 12 items (GHQ-12) was *M* = 6.16 (SD = 3.76), with a reliability coefficient of the optimal measurement scale of Cronbach α = 0.910. PD is more present among women, OR = 1.916, 95% CI = 1.613–2.273; aged 29 or younger, OR = 2.651, 95% CI = 2.219–3.168; without a couple OR = 1.959, 95% CI = 1.643–2.336; with lower educational level (secondary school or lower), OR = 2.132, 95% CI = 1.771–2.567; living in a house without balcony/terrace/yard/garden, OR = 1.631, 95% CI = 1.232–2.160; without children, OR = 2.222, 95% CI = 1.873–2.639; and not being a HCWs, OR = 1.385, 95% CI = 1.062–1.580. Higher PD was found among public employees (78.7%) than among workers of private companies (70.6%) and self-employed workers (61.8%), *P* < .001.	6/8
Ruiz-Frutos et al^[55]^ Peru	April–September 2020	To analyze the influence of the sources used by the population in Peru to obtain information on COVID-19 and its association with developing PD and preventive measures against contagion.	Cross-sectional	1699 participants	GHQ-12	In a first analysis, there were 1699 cases, of which 59.68% (1014) had PD. The percentage of people with PD when the degree of concern is below the cutoff point is 40.84%. However, this percentage increases to 65.99% when it exceeds that point. In the latter branch, PD cases can be classified according to *confidence in the health system* to diagnose and recognize COVID-19; a confidence of <6.5 points would increase PD cases to 71.66%, compared to the 55.90% of cases found when confidence in the health system is higher.	8/8
Alvarado et al^[18]^ Chile	May–July 2020	To evaluate HCWs’ mental health and its associated factors during the pandemic in Chile.	Cross-sectional	1934 HCWs	GHQ-12 PHQ-9 C-SSRS	The GHQ-12 total score for the whole group had a mean of 16.3 and a SD of 6.3 points; a range between 1 and 36 points, and a distribution resembling a normal curve, but asymmetric to the right (skewness: 0.376) and flattened at the peak (kurtosis: −0.349) was identified. When compared by sex, women had a significantly higher score than men, and when compared by place of work, no significant differences were found. When used as a screening tool for mental disorder, 58.9% of respondents were found to be positive (score of 5 or more points).	6/8
Yélamos Agua et al^[56]^ Spain	March–April 2020	To assess the effects of the COVID-19 on the physical and mental health of non-HCWs.	Cross-sectional	1089 participants	UWES-9 SOC-13 GHQ-12	At low levels of WE, the percentage of distress is higher (77.9%). Low levels of SOC correspond to the highest percentages of distress (86.3%). The 94.1% believe it necessary for professionals and volunteers involved in COVID-19 to receive psychological support.	8/8
Ruiz-Frutos et al^[14]^ Spain	March–April 2020	To study the differences between those who work away from home and those who do so from home, when the effects of fear of contagion cross with those of confinement, about the PD during the COVID-19 in Spain.	Cross-sectional	1089 non-HCWs	GHQ-12	65.1% of all non-HCWs had PD (GHQ-12 cut point ≥3), with *M* = 4.51 (SD = 3.42), being slightly higher the percentage among those who worked away from home (67.3%), with *M* = 4.61 (SD = 3.39) than those who worked from home *M* = 4.44 (SD = 3.45), while not being this a statistically significant difference.	6/8
López-Atanes et al^[57]^ Spain	April 2020	To identify the psychosocial impact of the pandemic-related lockdown on cancer patients, and thus make a first exploratory and empirical approximation to distress levels and related factors in such a vulnerable group.	Cross-sectional	2779 cancer patients	K-6	33.5% of patients yield scores above the cutoff point defined above in the K-6 scale,^[13]^ suggestive of a pathological state of anxiety or depression. At the time the survey was undertaken, only 1.5% of patients had been diagnosed with COVID-19, although a further 4.9% believed that they had or had COVID-19-like symptoms.	8/8
Gómez-Salgado et al^[58]^ Spain	May–June 2020	To analyze from a gender perspective the PD experienced by the medical workforce during the peak of the pandemic in Spain.	Cross-sectional	667 hospital workers	GHQ-28 PSS-14	The mean score on the GHQ-28 was above the cutoff of 4/5 in women and men (10.44 and 7.10, respectively) but was significantly higher in women (*P* < .01). Quality of health was analyzed using the GHQ-28 and the case rate was found to be significantly higher in women than in men (78.4% vs 61.3%, *P* < .001).	8/8
Parrado-González and León-Jariego^[59]^ Spain	March–April 2020	To describe the levels of PD and SOC of HCWs during the crisis caused by COVID-19, the relationship between both variables, and their health status.	Cross-sectional	1459 HCWs	SOC-13 GHQ-12	The mean score obtained on the scale total was 5.38, with a standard deviation of 2.99. Establishing a cutoff point of 3 or more points to assess the presence of psychological discomfort, the results showed that 80.6% of the HCWs who participated in this study had this psychic morbidity.	8/8
Leira-Sanmartín et al^[60]^ Spain	March–April 2020	To measure the psychological impact of the COVID-19 pandemic on the Spanish population and to identify risk and protective factors for this impact.	Cross-sectional	1596 participants	IES-R GHQ-12	The overall level of PD (GHQ-12) had a mean score of 3.44 (SD = 3.37), with 48.8% of participants with symptoms associated with mental health impairment (≥3 points).	6/8
Ruiz-Frutos et al^[61]^ Spain	June–July 2020	To analyze a group of variables (demographic, professional, health-related, working environment-related), measuring their association with the presence of psychological burden on the workforce.	Cross-sectional	657 workers	GHQ-12	84.2% of the sample exceeded the cutoff point (12 points or more total score, Likert scoring system) of the GHQ-12 test, suggesting the need to further explore the presence of any nonpsychotic mental disorder. The average Goldberg score in our sample was 16.8 (SD: 5.5). In addition, 81.6% were HCWs while 15.3 were the average years of professional experience (SD: 10.9).	6/8
Gómez-Salgado et al^[62]^ Spain	March–April 2020	To analyze whether the information the public receives about COVID-19, by surveying about information received about COVID-19 regarding its source, time, assessment, or the beliefs expressed in it, had an influence on the level of distress of non-HCWs in Spain.	Cross-sectional	1089 non-HCWs	GHQ-12	Among the 1089 respondents, 65.1% (709) showed PD. When analyzing the sample by gender, 71.6% of women and 52.4% of men showed PD. In addition, it is possible to observe a statistically significant association between gender and distress (*P* < .001). By age, the percentage of cases with PD was higher (69.4%) among the younger (under-median age, 43 years), than among the older (60.4%), and there was a statistically significant association between variables (*P* = .002). The association was also statistically significant between the type of home, with (75.2%) or without (24.8%) outdoor space, and distress (*P* = .025).	8/8
Gómez-Salgado et al^[63]^ Spain	April–June 2020	To know the influence that the history of contact with the SARS-COV-2 virus and the SOC have had in the development of PD in the collective of Occupational Physicians and Nurses in Spain during the first months of the pandemic.	Cross-sectional	499 occupational health workers	GHQ-12 SOC-13	The percentage of workers with high PD was higher in women (72.87%) than in men (51.46%); it was also higher in those aged 51 or younger (70.28%) than in those over the age of 51 (60.46%), and higher in public sector workers (72.68%) than those in the private sector (61.39%). No statistically significant difference has been observed between PD and educational level, living as a couple, having children, working away from home, having a pet, or between being a physician or a nurse.	6/8
Mediavilla et al^[64]^ Spain	March–April 2020	To analyze the PD in a Spanish population sample during the COVID-19 pandemic, identifying the predictive character and role that sociodemographic variables, the presence of physical symptoms, and other health-related variables may have.	Cross-sectional	4180 participants	GHQ-12	72% of participants had PD. The highest percentage of PD was observed among people who were working outside home (48.5%), and a low percentage of PD was observed among people living with children or youngsters under the age of 16 (49.7%). A greater presence of PD was observed in women (79.6%) and in persons of lower middle age (*M* = 39.03, SD = 12.42).	8/8
Domínguez-Salas et al^[65]^ Spain	April–June 2020	The objectives were: (1) to explore the frequency of perceived discrimination during the initial outbreak of the pandemic and (2) to examine the association between perceived discrimination and depression symptoms, PD, and death thoughts	Cross-sectional	2053 hospital workers	PHQ-9 GHQ-12 C-SSRS	Thirty percent of the respondents reported discrimination and/or stigmatization. Perceived discrimination was associated with higher depression (*B* = 2.4, 95% CI: 1.8–2.9) and PD (*B* = 1.1, 95% CI: 0.7–1.4) scores, and with a 2-fold increase in risk of reporting death thoughts (OR = 2.0, 95% CI: 1.4–3.1).	6/8
Ruiz-Frutos et al^[66]^ Spain	March–April 2020	To analyze PD in a sample of Spanish population, identifying the predictive nature of the information received, the preventive measures taken, level of concern, beliefs, and knowledge about the infection.	Cross-sectional	4180 participants	GHQ-12	Of the total participants, 71.98% presented PD. Taking into account the assessment of the amount of information available on COVID-19, the results showed statistically significant differences in symptoms (*t* = 3.025, *P* = .003, Cohen *d* = 0.097), preventive measures (*t* = 2.749, *P* = .006, Cohen *d* = 0.093), transmission routes (*t* = 2.487, *P* = .013, Cohen *d* = 0.085), prognosis (*t* = 5.415, *P* = .001, Cohen *d* = 0.189), and treatment (*t* = 4.379, *P* ≤ .001, Cohen *d* = 0.149), with negligible effect sizes. As predictive factors, the degree of concern for COVID-19 was identified (OR = 1.244, 95% CI = 1.179–1.312), the number of hours spent consulting information on COVID-19 (OR = 1.038, 95% CI = 1.009–1.068), or the need for psychological support (OR = 1.135, 95% CI = 1.094–1.177)	8/8
Gómez-Salgado et al^[67]^ Peru	April–September 2020	The effects that COVID-19 had on citizens of Peru are described.	Cross-sectional	1699 participants	GHQ-12	With a cutoff point of GHQ-12 ≥ 3, 59.7% of globally studied cases have PD. The overall mean score on the 12 items (GHQ-12) is *M* = 4.18, SD = 3.52.	8/8
Gómez-Salgado et al^[68]^ Spain	March–April 2020	To describe the level of PD of active HCWs during the COVID-19 pandemic, and its relationship with PD according to the professional category.	Cross-sectional	1459 HCWs	GHQ-12 UWES-9	PD was reported by 80.6% of HCWs. WE as high with a total mean score of 5.04 (SD = 1.14). The results showed that distressed professionals showed significantly lower levels of WE.	8/8
Estrada-Araoz et al^[69]^ Spain	March–May 2022	To investigate disparities in the impact that the COVID-19 pandemic has had on the PD and WE of construction workers of both sexes in southern Spain and to correlate these impacts with sociodemographic and health-related factors.	Cross-sectional	857 construction workers	EIQ COVID-19 GHQ-12 UWES-9	Overall, 29.2% of respondents had PD (GHQ > 3), with statistically significant differences by sex, that is, higher among women (37.7%) than among men (27.3%) (*P* = .37). In terms of marital status, among those without a partner, women (43.8%) were more likely to have PD than men (31.1%) (*P* = .031).	6/8
Canal-Rivero et al^[70]^ Peru	July–August 2022	To determine whether or not PD is significantly related to burnout syndrome in regular basic education teachers upon their return to face-to-face classes.	Cross-sectional	351 regular basic education teachers	K-10 MBI	The level of PD of 40.7% of the teachers was moderate, 35.3% was low, and 24% was high. These results indicate that the teachers showed feelings of sadness, uncertainty, confusion, worry, and hopelessness upon returning to face-to-face classes.	8/8
Torres-Martín et al^[71]^ Spain	May–November 2020	To explore the impact of COVID-19 as well as possible gender differences on mental health status and suicidality in a cohort of HCWs.	Longitudinal	1432 HCWs	GHQ-28	HCWs informed of a worsening in somatic symptomatology over the follow up period. Gender differences were found in all GHQ-28 dimensions as well in the total score of the questionnaire. Post hoc analyses displayed significant interaction between the time and gender in somatic and anxiety dimensions as well as in GHQ-28 total score.	9/11
López Steinmetz et al^[17]^ Argentina	March–June 2020	To analyze differences in general MHS indicators, by the (1) sites of residence with different prevalence of COVID-19 cases, and (2) quarantine duration; (3) to assess multiple relationships between each general MHS indicator and potentially affecting factors.	Cross-sectional	5013 women	GHQ-12 K-10	Worse self-perceived health and higher psychological discomfort affected significantly more women residing in sites with high prevalence of COVID-19 cases, compared to those residing in sites with intermediate prevalence, but effect sizes were small. Mean scores of all general MHS indicators were significantly worse for longer quarantine subperiods (up to 53, 68, and 80-day duration) than for shorter subperiods (up to 7, 13, and 25-day duration). Being a younger age, having mental disorder history, and longer quarantine durations were associated to worsening MHS, while the lack of previous suicide attempt has a protective effect.	8/8
Gómez-Salgado et al^[31]^ Argentina	March–May 2020	To find out if subjective temporality-time perspective and temporal focus- and self-control and impulsivity have repercussions on PD, particularly during the COVID-19 pandemic, in an Argentinian sample.	Cross-sectional	279 participants	ZTPI TFS BSCS CUBI-18 K-10	PD correlated statistically significantly with all variables except sensation seeking. The correlation was positive and significant with negative past, hedonistic present, fatalistic present, past temporal focus, future temporal focus, compulsive urgency, and impulsivity due to unpredictability. In contrast, PD was negatively and significantly related to positive past, future, present temporal focus, and self-control. The direct relationship between PM and negative past, and the inverse relationship between PD and self-control with large effect sizes are highlighted.	8/8
Liebana-Presa et al^[72]^ Spain	2020–2021 basket season	To determine the risk of PD in basketball coaches analyzing psychosocial risk factors that may influence their sports work during the 2020 to 2021 season characterized by COVID-19 pandemic conditions.	Cross-sectional	94 basketball coaches	K-10 Psychosocial Risk Assessment at Work. ISTAS21	The results indicate that the asymmetry was positive in dimensions 2, 4, and 6, which shows that there was a greater concentration of such responses as “many times” or “always”; on the contrary, in dimensions 1, 3, and 5, the response options were concentrated around “sometimes” or “never.” The participants were grouped into 3 grades: grade 1 corresponds to low PD in coaches, that is, the participants’ means were below the 25th percentile (*x* ≤ 12); grade 2, moderate level of PD, the means are between the 26th and 74th percentiles (*x* ≥ 13 to *x* ≤ 16); finally, grade 3, coaches with high levels of PD, is characterized by the means above the 75th percentile (*x* ≥ 17).	8/8
López-Gutiérrez et al^[73]^ Spain	September 2021–June 2022	To describe self-perceived health status, general stress, and prenatal stress and to analyze relations and associations with sociodemographic factors.	Cross-sectional	297 women in their first trimester of pregnancy	PDQ PSS GHQ-28	Of the participating women, 6% presented somatic symptoms according to the GHQ-28 scale, and all of them belong to the multiparous group. Regarding the anxiety/insomnia variable, 18% of the women scored positively, with a higher percentage in multiparous women than in primiparous women (24.7% vs 11.1%).	6/8
Gorbeña et al^[74]^ Spain	April 2020	To compare the state of psychological discomfort and stress during this period of confinement, of a group of people, based on their level of sporting activity and sex.	Cross-sectional	660 athletes and nonathletes	K-10	When analyzing the PD of the participants (n = 660), it was found that 30% (n = 198) reported being in a situation of “extreme discomfort,” 24% (n = 161) were in “moderate discomfort,” 27.3% (n = 180) were in “mild discomfort,” and only 18.3% (n = 121) reported “no discomfort at all.” This means that more than half of the sample (54.4%) was in a state of PD to be considered. When relating PD to sex, it can be seen that of the total sample that reported being in a situation of extreme PD (n = 198), 40.5% (n = 122) were women and 21.2% (n = 76) were men. Among professional athletes, 33% (n = 31) had no PD, 37.3% (n = 44) were in the category of mild discomfort, 22.9% (n = 27) had moderate discomfort, and 11.9% (n = 14) had extreme discomfort. This is a low percentage compared to 26.2% (n = 94) of amateur sportsmen and women or 49.2% (n = 90) of nonathletes.	6/8
Benítez et al^[75]^ Spain	Pre/post COVID-19	(1) To explore the psychological effects of the pandemic in a sample of young adults, and (2) to analyze differences, comparing data from a previous intervention conducted last year (a non-pandemic situation) with the same type of population.	Longitudinal	151 students	SWLS SPANE Psychological Well-being. Social Well-being. GHQ-12	As for the PD assessed with the GHQ-12, changes in the scores of the 3 groups are shown. As with positive mental health, the average score of participants in the COVID intervention group shows a significant decrease in symptomatology and an increase for the other 2 groups, though small (0.15) and nonsignificant for the pre-COVID intervention group. All effects have resulted statistically significant (between groups *F* = 9.38, *P* = .042, η^2^ = 0.042; within groups *F* = 4.37, *P* = .030, η^2^ = 0.031, and interaction effect *F* = 10.15, *P* < .001, η^2^ = 0.121).	9/11

AIS-8 = Athens Insomnia Scale, AMICO = Anxiety and Fear of COVID-19, APGAR = Adaptation, Partnership, Growth, Affection, Resolve, BIT = Brief Inventory of Thriving, BRS = Brief Resilience Scale, BSCS = Brief Self-Control Scale, CD-RISC10 = the short version of the Connor–Davidson Resilience Scale, CI = confidence interval, COPE-28 = Coping Orientations to Problems Experienced , C-SSRS = Columbia Suicide Severity Rating Scale, CUBI-18 = Urgency, Sensation Seeking and Impulsivity Questionnaire, DTS-8 = Davidson Trauma Scale, EBS = Emotional Burnout Scale, ECT = Workload Scale, EIQ COVID-19 = Emotional Impact Questionnaire COVID-19, EPPC-Cov19 = COVID-19 Contagion Concern Scale, ERQ = Emotion Regulation Questionnaire, GHQ-12 = General Health Questionnaire, 12 items, GHQ-28 = General Health Questionnaire, 28 items, HCWs = healthcare workers, IES-R = Impact of Event Scale – Revised, K-10 = Kessler Psychological Distress Scale, 10 items, K-6 = Kessler Psychological Distress Scale, 6 items, *M* = mean, MBI = Maslach Burnout Inventory, MHS = Mental Health Status, MOS = Medical Outcomes Study, OR = odds ratio, PD = psychological distress, PDQ = Prenatal Distress Questionnaire, PHQ-9 = Patient Health Questionnaire 9, PSS = Perceived Stress Scale, PTSD = posttraumatic stress disorder, PVDQ = Perceived Vulnerability to Disease Questionnaire, RSES = Rosenberg Self-Esteem Scale, SCL-90-R = Symptom Check-List-90 Revised, SD = standard deviation, SOC = Sense of Coherence, SOC-13 = Sense of Coherence Scale, 13 items, SPANE = Scale of Positive and Negative Experience, SSS = Social Support Scale, SWLS = Satisfaction with Life Scale, TFS = Temporal Focus Scale, UWES-9 = Utrecht Work Engagement Scale, 9 items, WE = work engagement, ZTPI = Zimbardo Time Perspective Inventory.

A meta-analysis of the prevalence of PD in Spanish-speaking population groups during the COVID-19 pandemic was conducted with the studies that included the necessary information (n = 35). The samples affected by PD in each study and group (general population n = 19, workers n = 8, and healthcare workers n = 8), independently of the scale, were included in the meta-analysis. The total sample of the general population in the meta-analysis was n = 30,572 with a prevalence value of 49% (95% CI 40%–58%). The total sample of the healthcare workers in the meta-analysis was n = 6448 with a prevalence value of 56% (95% CI 43%–68%). The total sample of the workers in the meta-analysis was n = 8831 with a prevalence value of 63% (95% CI 51%–73%).

**Figure 2.**
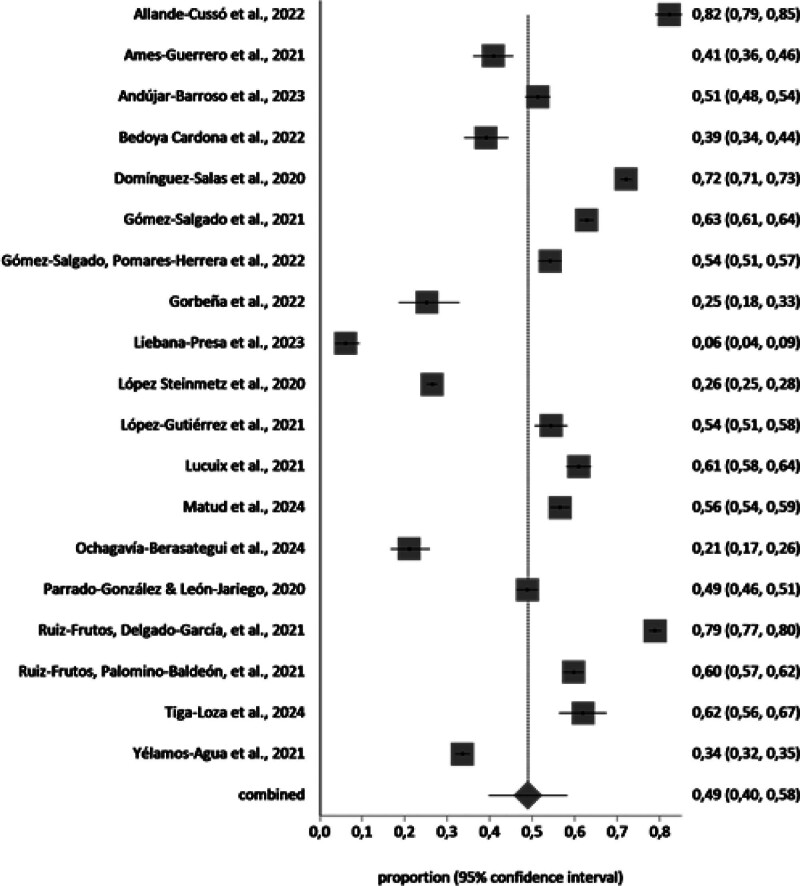
Proportion meta-analysis plot: general population (random effects).

**Figure 3.**
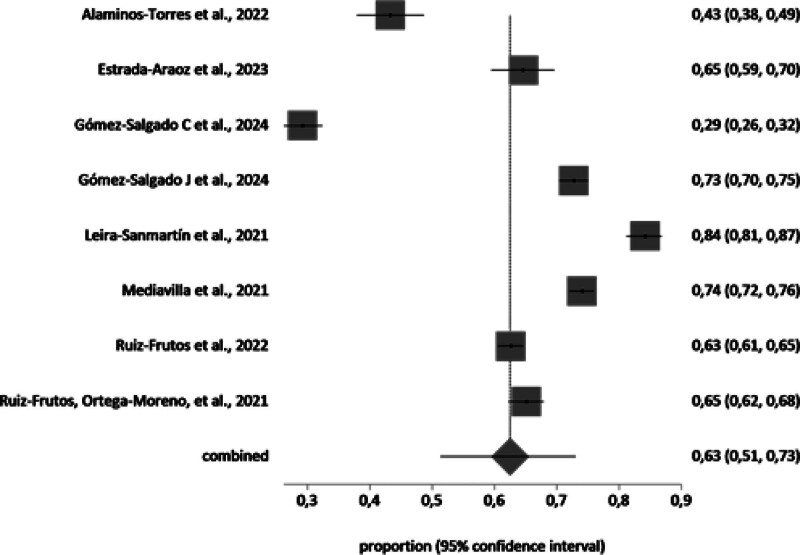
Proportion meta-analysis plot: workers (random effects).

**Figure 4.**
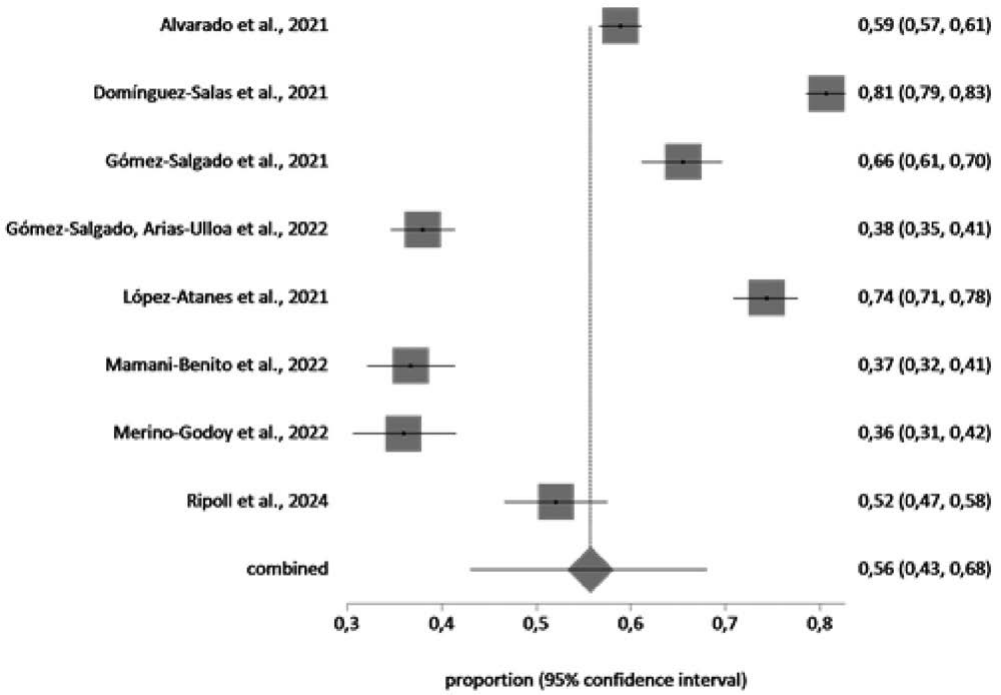
Proportion meta-analysis plot: healthcare workers (random effects).

The forest plot is shown in Figures 2–4. Random effects were used because the *I*^2^ is high. The *I*^2^ value was 99.6%, 99%, and 99,1% for the general population, healthcare workers, and workers, respectively, and Egger test value showed no publication bias.

## 4. Discussion

The COVID-19 pandemic has had a significant impact on mental health in different Spanish-speaking countries, affecting a number of professions. Several methods of PD assessment have been used in these countries.

### 4.1. Comparison by country and profession

PD was well defined among the country populations in Spain. A high level of PD was observed among healthcare workers (74%), along with depressive signs (27%). This result highlights the need to secure proper access to personal protective equipment not just to safeguard the workers’ physical condition but also to preserve their mental condition.^[47]^ In addition, with 80.6% showing high levels of distress, a significant relationship was observed between PD and lower levels of WE. This suggests that PD may decrease workers’ motivation and WE with their tasks, potentially affecting the quality of care they are able to provide.^[67]^ This distress was not limited to healthcare workers, however, as 77.9% of non-healthcare workers reported symptoms of PD, with low levels of SOC strongly associated with high PD. These findings suggest that SOC may be an important protective factor through which individuals are able to better cope with stress and adversity (such as those encountered during the pandemic).^[14]^

In Peru,^[66]^ studies revealed that approximately 59.7% of the general population experienced PD during the pandemic, lower figures compared to other countries such as Spain, where a prevalence of 72% was reported.^[63]^ This difference could reflect variations in cultural and economic factors and in the perception of risk across countries. Despite rigorous compliance with preventive measures in Peru, the high COVID-19 case fatality rate in the country compared to other Latin American countries could be attributed to factors such as the precariousness of the health system and high levels of poverty, a situation that emphasizes the complexity of pandemic management in contexts with limited economic and health infrastructure.^[75]^ Moreover, health workers in the Puno region showed that concern about COVID-19 infection was a significant predictor of PD among health workers in Peru, a finding that underscores the importance of addressing fear of infection not only as a physical problem, but also as a psychological risk factor that affects the mental well-being of health workers.^[38]^ In Chile,^[4]^ PD levels were also high, with 78.83% of the general population showing significant symptoms. The combination of factors such as job stress and a low SOC increased these levels, particularly among women, where poor self-perception of health was strongly associated with high levels of PD. This finding emphasizes the relationship between how people perceive their own health and their mental well-being, which is crucial for designing intervention strategies that encompass the improvement of health perception as a key objective in the general population and, specifically, in healthcare workers.^[76]^

On the other hand, in Ecuador,^[28]^ 62.72% of the population experienced PE, with higher levels concentrated in women, people with higher education, and those who did not live with children under 16 years of age. These results are presented in a manner consistent with patterns observed in other Latin American countries^[66]^ and Spain,^[63]^ which raise the need to design specific interventions for these vulnerable groups. In Argentina,^[29]^ 60.9% of the sample was found to experience PD, and it was more prevalent among women and younger people. These findings support the trend observed in other contexts where women and young people appear to be more vulnerable to the psychological effects of the pandemic, possibly due to factors such as the additional burden of domestic responsibilities and the impact of social isolation.^[77]^

In Peru,^[69]^ basic education teachers reported 40.7% of moderate PD, during the return to face-to-face classes, and a significant and direct correlation was found between PD and burnout syndrome among these teachers. This relationship presents how chronic stress and prolonged emotional demands in the educational environment can cause severe emotional exhaustion, which in turn aggravates PD.^[78]^

Compared to non-Spanish-speaking countries, some differences have been found. In the case of a study conducted on healthcare workers in the United States,^[79]^ a 30% prevalence was observed for high and very high stress levels. In another study conducted among healthcare workers in Thailand,^[80]^ 41.97% were found to have PD symptoms. In these 2 examples, the values were slightly lower than those found in our meta-analysis (30% and 41.97%, vs 56%). Compared to the general population, higher rates are observed than in the case of health workers. In fact, in a study conducted in Iraq,^[81]^ it was observed that the prevalence of moderate and high perceived stress was 59.6% and 16.6%, respectively. Somewhat lower values were found in a study conducted in the general population in China (24.4%).^[82]^ In the present meta-analysis, the calculated prevalence is centered between the 2 values. This variability in results may be explained by differences in samples, data collection periods, measures adopted by governments, among others.

### 4.2. Comparison by assessment methods

The General Health Questionnaire (GHQ) is one of the most widely used instruments to assess PD, identifying mental health problems in the general population, particularly those related to nonpsychotic psychiatric disorders. This questionnaire, in its different versions such as the GHQ-12 or the GHQ-28, was widely used in several of the studies included in this review. The GHQ-12 was one of the most widely used (37 studies), as observed in studies in Spain, Chile, Peru, Ecuador, and Argentina. This questionnaire was effective in identifying levels of PD in different population groups, for example, among healthcare workers^[52]^ and non-healthcare workers.^[55]^ Along with other questionnaires, it helped to identify that workers with low levels of SOC and high levels of stress were more likely to experience PD.^[4,18,31]^ Also, in other studies (5 studies), the GHQ-28 was used in healthcare workers in Spain,^[13,57,70]^ including a specific item to assess suicidal thoughts, which allowed researchers to monitor this critical aspect of mental well-being. Although the study found a low prevalence of suicidal thoughts, the inclusion of this item highlighted the importance of ongoing mental health surveillance in situations of crisis and in the general population. In Peru,^[45]^ together with the Coping Orientations to Problems Experienced, this tool was used to explore how active and passive coping strategies were related to PD levels. Results showed that passive strategies, such as disengagement and emotional venting, were most associated with high levels of PD.^[83]^

Other instruments, such as the K-10 and K-6 (Kessler Psychological Distress Scale), allow a quick and effective assessment of the risk depression or anxiety disorders.^[84]^ The K-10 was used in studies with nursing students,^[48,69]^ in the general population,^[30]^ for studies on orthodontic patients,^[40]^ in the general Spanish population,^[42]^ in basketball coaches in a specific area,^[71]^ and in athlete/nonathlete comparison studies.^[73]^ The K-6 questionnaire was used to evaluate cancer patients in one study^[56]^ and to evaluate PD in healthcare workers during the COVID-19 pandemic.^[11,38]^

The 90-symptom checklist questionnaire (SCL-90-R) is a tool that measures a wide range of psychological symptoms and is specifically useful for identifying patterns of emotional and PD in various population settings. A study with a sample of Colombian adults,^[33]^ also found that females, young people under 35 years of age, and people who live unaccompanied had higher scores on this questionnaire, which could indicate a higher PE compared to other demographic groups. These results show how specific demographic factors can significantly influence the experience of PD during crisis situations.

The SCL-90-R is more detailed and covers a wider range of symptoms compared to other instruments such as the GHQ-12 or the K-10, which are more specific and concise. In fact, one study used the 2 tools mentioned above.^[17]^ The capacity of the SCL-90-R to measure multiple dimensions of PD makes it particularly useful in studies where a comprehensive assessment of psychological symptoms is sought. This allows for a more complete understanding of how different aspects of distress manifest themselves in the population.^[33]^

The results of these studies also have significant implications to help in the development of public policies and to design specific interventions. For example, the high prevalence of PD among women, youth, and healthcare workers suggests that it is important to prioritize these groups in the allocation of resources and the design of mental health. For instance, in migrant communities, it may be crucial to offer care in multiple languages, while in rural areas, telepsychology or trained community agents may be more appropriate. Furthermore, the contextual differences observed between countries highlight the need for culturally adapted interventions, which take into account factors such as the infrastructure of health settings and government responses. The results can also guide the development of awareness campaigns to reduce the stigma associated with seeking psychological help, particularly in vulnerable populations such as immigrants and marginalized communities. In this case, involving public figures or local leaders may also be useful in increasing acceptance and the overall impact of these initiatives.

## 5. Limitations

The studies included in this review were conducted in different countries with diverse cultural contexts, health systems, and socioeconomic levels, and this variability may hinder direct comparison of results, as cultural and contextual factors significantly influence the experience of PD and the responses of the population groups. Countries adopted different measures to cope with the pandemic (such as quarantines, school closures, and economic support policies), which directly influenced the level of PD of the population. This review may be limited by the inability to isolate the impact of these policies when comparing results across countries. Also, the included studies used different measurement instruments to assess PD (e.g. GHQ-12, GHQ-28, K-6, K-10, SCL-90-R), so this diversity of tools may lead to problems of comparability, as each instrument has different scales, cutoff points, and sensitivity for detecting PD.

Publication bias may represent a limitation of this review, since studies with negative or nonsignificant results may be underrepresented in the databases consulted. This phenomenon could lead to an overestimation of the prevalence of PD in the contexts analyzed. To try to mitigate this bias in future research, it would be important to implement strategies such as including regional databases, as well as performing publication bias analysis using funnel plots and specific statistical tests.

## 6. Conclusions

Through the analysis of studies carried out in Spain, Peru, Chile, Ecuador, Argentina, and Colombia, it has evidenced that PD has manifested itself in a significant and heterogeneous way, affecting both the general population and specific professional groups, such as health workers, teachers, and other essential workers.

The differences observed across countries and professions highlight the influence that sociocultural, economic, and occupational factors have on the experience of PD. Also, the studies have underlined the importance of the SOC and WE as protective factors against PD, especially in challenging work environments.

All together, these findings underscore the urgent need to develop mental health interventions that are culturally sensitive and responsive to the specific needs of different population groups in Spanish-speaking countries. The COVID-19 pandemic has exacerbated existing inequalities and revealed the importance of an integrated and contextualized approach to mental health care. Public policies must be developed that give greater priority to equal access to hospital mental health services and that the response capacity in this type of crisis context is strengthened, which would help minimize the impact of PD on the most vulnerable populations. Likewise, regional cooperation programs or strategies should be implemented, so that resources and best practices can be shared between Spanish-speaking countries, in turn, collective efforts could also be optimized to address similar challenges in the future.

It would be essential to strengthen the validity and comparability of the results for future research by adopting more standardized methodologies. This should include a review of the use of a single validated instrument to assess PD in different contexts. Taking as an example, questionnaires such as the GHQ-12 or the K-10 could be adopted to help make more precise comparisons of prevalence between these countries and population groups, helping to achieve a comprehensive understanding of the phenomenon and facilitating the design of interventions evidence-based.

## Author contributions

**Conceptualization:** Kenny Escobar-Segovia, Sara Domínguez-Salas, Juan Jesús García-Iglesias, Daniel López-López, Regina Allande-Cussó, Juan Gómez-Salgado.

**Data curation:** Sara Domínguez-Salas, Carlos Ruiz-Frutos, Alejandro Artero-García.

**Formal analysis:** Kenny Escobar-Segovia, Juan Jesús García-Iglesias, Regina Allande-Cussó, Carlos Ruiz-Frutos, Alejandro Artero-García.

**Funding acquisition:** Daniel López-López, Juan Gómez-Salgado.

**Investigation:** Sara Domínguez-Salas, Daniel López-López, Carlos Ruiz-Frutos, Juan Gómez-Salgado.

**Methodology:** Kenny Escobar-Segovia, Juan Jesús García-Iglesias, Regina Allande-Cussó, Alejandro Artero-García.

**Project administration:** Sara Domínguez-Salas, Carlos Ruiz-Frutos, Juan Gómez-Salgado.

**Resources:** Kenny Escobar-Segovia, Juan Jesús García-Iglesias, Daniel López-López, Carlos Ruiz-Frutos, Alejandro Artero-García, Juan Gómez-Salgado.

**Software:** Juan Jesús García-Iglesias, Daniel López-López, Carlos Ruiz-Frutos, Juan Gómez-Salgado.

**Supervision:** Sara Domínguez-Salas, Juan Jesús García-Iglesias, Regina Allande-Cussó, Carlos Ruiz-Frutos, Juan Gómez-Salgado.

**Validation:** Juan Jesús García-Iglesias, Daniel López-López, Alejandro Artero-García.

**Visualization:** Sara Domínguez-Salas, Juan Jesús García-Iglesias, Daniel López-López, Regina Allande-Cussó, Carlos Ruiz-Frutos, Juan Gómez-Salgado.

**Writing—original draft:** Kenny Escobar-Segovia, Juan Jesús García-Iglesias, Alejandro Artero-García.

**Writing—review & editing:** Sara Domínguez-Salas, Daniel López-López, Regina Allande-Cussó, Carlos Ruiz-Frutos, Juan Gómez-Salgado.

## Supplementary Material


