# Supplementary material for: Psychological distress in Spanish-speaking countries during the COVID-19 pandemic: A systematic review and meta-analysis
Source: Medicine (Baltimore). 2026 Jan 9;105(2):e47062. doi: 10.1097/MD.0000000000047062 (PMC12794965; doi:10.1097/MD.0000000000047062)
Supplement: Supplementary file 1 [file medi-105-e47062-s001.docx]

**Table S1. Scores of cross-sectional observational studies.**

| **Study** | **JBI** | **The participants and the environment are described in detail** | **Inclusion criteria are clearly defined** | **Exposure was validly and reliably measured** | **The criterion used to measure the condition was objective** | **Confounding factors were identified** | **Strategies for dealing with confounding factors** | **Results were validly and reliably measured** | **Appropriate statistical analysis was used** |
| --- | --- | --- | --- | --- | --- | --- | --- | --- | --- |
| (J. Gómez-Salgado, Carrión-Rico, et al., 2024) | 6/8 | Yes | Yes | Yes | Yes | No | No | Yes | Yes |
| (Pilar Matud et al., 2023) | 8/8 | Yes | Yes | Yes | Yes | Yes | Yes | Yes | Yes |
| (Ripoll et al., 2024) | 6/8 | Yes | Yes | Yes | Yes | No | No | Yes | Yes |
| (Tiga-Loza et al., 2024) | 7/8 | Yes | Yes | Yes | Yes | Yes | No | Yes | Yes |
| (Carranza Esteban et al., 2023) | 6/8 | Yes | Yes | Yes | Yes | No | No | Yes | Yes |
| (C. Gómez-Salgado, Camacho-Vega, Allande-Cussó, Ruiz-Frutos, Ortega-Moreno, Martín-Pereira, et al., 2024) | 6/8 | Yes | Yes | Yes | Yes | No | No | Yes | Yes |
| (Ochagavía-Berasategui et al., 2024) | 6/8 | Yes | Yes | Yes | Yes | No | No | Yes | Yes |
| (J. Gómez-Salgado, Delgado-García, et al., 2024) | 6/8 | Yes | Yes | Yes | Yes | No | No | Yes | Yes |
| (Andújar-Barroso et al., 2023) | 8/8 | Yes | Yes | Yes | Yes | Yes | Yes | Yes | Yes |
| (Dorado Barbé et al., 2023) | 8/8 | Yes | Yes | Yes | Yes | Yes | Yes | Yes | Yes |
| (Matud et al., 2024) | 6/8 | Yes | Yes | Yes | Yes | No | No | Yes | Yes |
| (J. Gómez-Salgado, Arias-Ulloa, et al., 2022) | 8/8 | Yes | Yes | Yes | Yes | Yes | Yes | Yes | Yes |
| (Ames-Guerrero et al., 2021) | 6/8 | Yes | Yes | Yes | Yes | No | No | Yes | Yes |
| (Ruiz-Frutos, Adanaqué-Bravo, et al., 2022) | 8/8 | Yes | Yes | Yes | Yes | Yes | Yes | Yes | Yes |
| (Alaminos-Torres et al., 2022) | 8/8 | Yes | Yes | Yes | Yes | Yes | Yes | Yes | Yes |
| (Mediavilla, Fernández-Jiménez, Martínez-Alés, et al., 2021) | 8/8 | Yes | Yes | Yes | Yes | Yes | Yes | Yes | Yes |
| (Merino-Godoy et al., 2022) | 8/8 | Yes | Yes | Yes | Yes | Yes | Yes | Yes | Yes |
| (Ortiz-Calvo, Martínez-Alés, Mediavilla, González-Gómez, Fernández-Jiménez, Bravo-Ortiz, Moreno-Küstner, et al., 2022) | 8/8 | Yes | Yes | Yes | Yes | Yes | Yes | Yes | Yes |
| (Domínguez-Salas et al., 2021) | 6/8 | Yes | Yes | Yes | Yes | No | No | Yes | Yes |
| (Ruiz-Frutos, Ortega-Moreno, Soriano-Tarin, et al., 2021) | 8/8 | Yes | Yes | Yes | Yes | Yes | Yes | Yes | Yes |
| (Bedoya Cardona et al., 2022) | 6/8 | Yes | Yes | Yes | Yes | No | No | Yes | Yes |
| (J. Gómez-Salgado, Adanaque-Bravo, et al., 2021) | 6/8 | Yes | Yes | Yes | Yes | No | No | Yes | Yes |
| (Lucuix et al., 2021) | 6/8 | Yes | Yes | Yes | Yes | No | No | Yes | Yes |
| (Mamani-Benito et al., 2022) | 6/8 | Yes | Yes | Yes | Yes | No | No | Yes | Yes |
| (Martínez-Caballero et al., 2021) | 8/8 | Yes | Yes | Yes | Yes | Yes | Yes | Yes | Yes |
| (Allande-Cussó et al., 2022) | 8/8 | Yes | Yes | Yes | Yes | Yes | Yes | Yes | Yes |
| (Ruiz-Frutos, Delgado-García, Ortega-Moreno, Duclos-Bastías, Escobar-Gómez, et al., 2021) | 8/8 | Yes | Yes | Yes | Yes | Yes | Yes | Yes | Yes |
| (J. Gómez-Salgado, Palomino-Baldeón, et al., 2022) | 8/8 | Yes | Yes | Yes | Yes | Yes | Yes | Yes | Yes |
| (Alvarado et al., 2021) | 6/8 | Yes | Yes | Yes | Yes | No | No | Yes | Yes |
| (Ruiz-Frutos, Ortega-Moreno, Allande-Cussó, Ayuso-Murillo, et al., 2021) | 8/8 | Yes | Yes | Yes | Yes | Yes | Yes | Yes | Yes |
| (Ruiz-Frutos, Ortega-Moreno, Allande-Cussó, et al., 2020) | 6/8 | Yes | Yes | Yes | Yes | No | No | Yes | Yes |
| (Yélamos Agua et al., 2021) | 8/8 | Yes | Yes | Yes | Yes | Yes | Yes | Yes | Yes |
| (López-Atanes et al., 2021) | 8/8 | Yes | Yes | Yes | Yes | Yes | Yes | Yes | Yes |
| (Gómez-Salgado et al., 2020) | 8/8 | Yes | Yes | Yes | Yes | Yes | Yes | Yes | Yes |
| (Parrado-González & León-Jariego, 2020) | 6/8 | Yes | Yes | Yes | Yes | No | No | Yes | Yes |
| (Leira-Sanmartín et al., 2021) | 6/8 | Yes | Yes | Yes | Yes | No | No | Yes | Yes |
| (Ruiz-Frutos, Ortega-Moreno, Dias, et al., 2020) | 8/8 | Yes | Yes | Yes | Yes | Yes | Yes | Yes | Yes |
| (J. Gómez-Salgado, Ortega-Moreno, et al., 2021) | 6/8 | Yes | Yes | Yes | Yes | No | No | Yes | Yes |
| (J. Gómez-Salgado, Andrés-Villas, et al., 2020) | 8/8 | Yes | Yes | Yes | Yes | Yes | Yes | Yes | Yes |
| (Mediavilla, Fernández-Jiménez, Andreo, et al., 2021) | 6/8 | Yes | Yes | Yes | Yes | No | No | Yes | Yes |
| (Domínguez-Salas et al., 2020) | 8/8 | Yes | Yes | Yes | Yes | Yes | Yes | Yes | Yes |
| (Ruiz-Frutos, Palomino-Baldeón, Ortega-Moreno, Villavicencio-Guardia, Dias, et al., 2021) | 8/8 | Yes | Yes | Yes | Yes | Yes | Yes | Yes | Yes |
| (Gómez‐Salgado et al., 2021) | 8/8 | Yes | Yes | Yes | Yes | Yes | Yes | Yes | Yes |
| (C. Gómez-Salgado, Camacho-Vega, Allande-Cussó, Ruiz-Frutos, Ortega-Moreno, Linares-Manrique, et al., 2024) | 6/8 | Yes | Yes | Yes | Yes | No | No | Yes | Yes |
| (Estrada-Araoz et al., 2023) | 8/8 | Yes | Yes | Yes | Yes | Yes | Yes | Yes | Yes |
| (López Steinmetz et al., 2020) | 8/8 | Yes | Yes | Yes | Yes | Yes | Yes | Yes | Yes |
| (Germano & Brenlla, 2023) | 8/8 | Yes | Yes | Yes | Yes | Yes | Yes | Yes | Yes |
| (Torres-Martín et al., 2021) | 8/8 | Yes | Yes | Yes | Yes | Yes | Yes | Yes | Yes |
| (Liebana-Presa et al., 2023) | 6/8 | Yes | Yes | Yes | Yes | No | No | Yes | Yes |
| (López-Gutiérrez et al., 2021) | 6/8 | Yes | Yes | Yes | Yes | No | No | Yes | Yes |
|  |  |  |  |  |  |  |  |  |  |

JBI: Joanna Briggs Institute Critical Appraisal Tool score

**Table S2. Scores of longitudinal or cohort studies.**

| Study | JBI | Were the two groups similar and recruited from the same population? | Were exposures measured in a similar way to assign people to both exposed and unexposed groups? | Was exposure measured in a valid and reliable way? | Were confounding factors identified? | Were strategies to address confounding factors indicated? | Were the groups/participants free of the outcome at the start of the study (or at the time of exposure)? | Were the results measured in a valid and reliable way? | Was the follow-up time reported and was it sufficient to produce results? | Was follow-up completed and, if not, were the reasons for missing follow-up described and explored? | Were strategies to address incomplete monitoring used? | Was an appropriate statistical analysis used? |
| --- | --- | --- | --- | --- | --- | --- | --- | --- | --- | --- | --- | --- |
| (J. Gómez-Salgado, Pomares-Herrera, et al., 2022) | 8/11 | Yes | Yes | Yes | Yes | No | Yes | Yes | Yes | No | No | Yes |
| (Canal-Rivero et al., 2022) | 9/11 | Yes | Yes | Yes | Yes | Yes | Yes | Yes | Yes | No | No | Yes |
| (Gorbeña et al., 2022) | 9/11 | Yes | Yes | Yes | Yes | No | Yes | Yes | Yes | Yes | No | Yes |

JBI: Joanna Briggs Institute Critical Appraisal Tool score
